# Alpha radiation from polymetallic nodules and potential health risks from deep-sea mining

**DOI:** 10.1038/s41598-023-33971-w

**Published:** 2023-05-17

**Authors:** Jessica B. Volz, Walter Geibert, Dennis Köhler, Michiel M. Rutgers van der Loeff, Sabine Kasten

**Affiliations:** 1 Alfred Wegener Institute Helmholtz Centre for Polar and Marine Research, Am Handelshafen 12, 27570 Bremerhaven, Germany; 2 Faculty of Geosciences, University of Bremen, Klagenfurter Strasse 4, 28359 Bremen, Germany; 3 MARUM - Center for Marine Environmental Sciences, Klagenfurter Strasse 4, 28359 Bremen, Germany

**Keywords:** Geochemistry, Marine chemistry, Environmental impact

## Abstract

In search for critical elements, polymetallic nodules at the deep abyssal seafloor are targeted for mining operations. Nodules efficiently scavenge and retain several naturally occurring uranium-series radioisotopes, which predominantly emit alpha radiation during decay. Here, we present new data on the activity concentrations of thorium-230, radium-226, and protactinium-231, as well as on the release of radon-222 in and from nodules from the NE Pacific Ocean. In line with abundantly published data from historic studies, we demonstrate that the activity concentrations for several alpha emitters are often higher than 5 Bq g^−1^ at the surface of the nodules. These observed values can exceed current exemption levels by up to a factor of 1000, and even entire nodules commonly exceed these limits. Exemption levels are in place for naturally occurring radioactive materials (NORM) such as ores and slags, to protect the public and to ensure occupational health and radiation safety. In this context, we discuss three ways of radiation exposure from nodules, including the inhalation or ingestion of nodule fines, the inhalation of radon gas in enclosed spaces and the potential concentration of some radioisotopes during nodule processing. Seen in this light, inappropriate handling of polymetallic nodules poses serious health risks.

## Introduction

Since the discovery of deep-sea polymetallic nodules, these mineral deposits have been studied for their commercial resource potential. The largest deposit of polymetallic nodules is found in the Clarion-Clipperton Zone (CCZ) in the NE Pacific Ocean at 4000–6000 m water depth, where approximately 30 billion tons (3 × 10^19^ g) of nodules cover vast areas of the seafloor^1^. The enrichment of critical elements such as Ni, Cu, Mn, Co, Li, REE, and Y within polymetallic nodules makes them appealing for commercial exploitation for the development of high technology (e.g., cell phones, batteries, permanent magnets)^2^. In times of an energy transition, the increase in the global demand for critical elements is striking. Although highly variable depending on the element, demand outlooks estimate a potential increase in the need for critical elements as much as six times by 2040^3^. Thus, the international interest in mining deep-sea minerals, has re-gained momentum^2^ and in 2021, Nauru triggered the so-called “2-year rule” (UNCLOS Agreement on Part XI)^4,5^ for polymetallic nodule mining in the CCZ, implying that commercial exploitation could already commence in 2023.

Many recent scientific studies have focused on the environmental impacts of future deep-sea mining at the deep seafloor^6,7^ while we present and discuss here the legal relevance of radioactive decay in polymetallic nodules for future nodule mining and processing as well as the arising health risks. We investigate the distribution of selected radioactive isotopes (Th-230, Ra-226, Pa-231, Rn-222) in polymetallic nodules from the CCZ. As these alpha-emitting radioisotopes and their daughter isotopes may constitute a substantial risk for humans when handling naturally occurring radioactive materials^8^ (NORM), we evaluate radiation safety aspects of polymetallic nodules, comparing them to other NORM and bringing them into context with existing NORM regulations. We contribute new radioisotope data of a set of nodules collected from different exploration areas in the CCZ and we build on a wealth of published radioisotope datasets from historic studies.

The radioisotopes Th-230 (half-life (t_1/2_) = 75,380 year) and Pa-231 (t_1/2_ = 32,760 year) are both produced in the ocean by the decay of dissolved U-238 and U-235, respectively. As Th-230 and Pa-231 are highly particle-reactive, both isotopes are scavenged from the water column and removed to the seafloor within decades and centuries, respectively^9^. Furthermore, Th-230 and Pa-231 as well as several of their daughter isotopes (e.g., Ra-226, actinium-227) are efficiently scavenged by and concentrated in iron-manganese oxides^10,11^, which make up the major fraction of polymetallic nodules. In the CCZ, sediments and nodules accumulate very slowly at rates of mm to cm/kyr and mm/Myr, respectively^12,13^. As a result, relatively high activities, particularly of Th-230 in substantial excess of its parent U-234 and its daughter isotope Ra-226 (t_1/2_ = 1620 year) occur in the sediments and nodules. To a lesser degree, Pa-231 in excess of its parent U-235 and its daughter isotopes like Ac-227 with decay chains dominated by alpha decays constitute further important sources of radiation in nodules and sediments. Thus, in contrast to most natural mineral deposits, where high activity concentrations are resulting from the in-situ decay of uranium (e.g., uranium ore), polymetallic nodules and deep-sea sediments have high inventories due to efficient scavenging of ions from the water column and adsorption of ions from the pore water. To our knowledge, this fact has never been considered as a radiation safety issue when handling and processing deep-sea sediments and nodules, also not in the wake of the rising deep-sea mining interest.

## Results and discussion

### Regulatory framework for naturally occurring radioactive materials (NORM)

**Figure 1:**
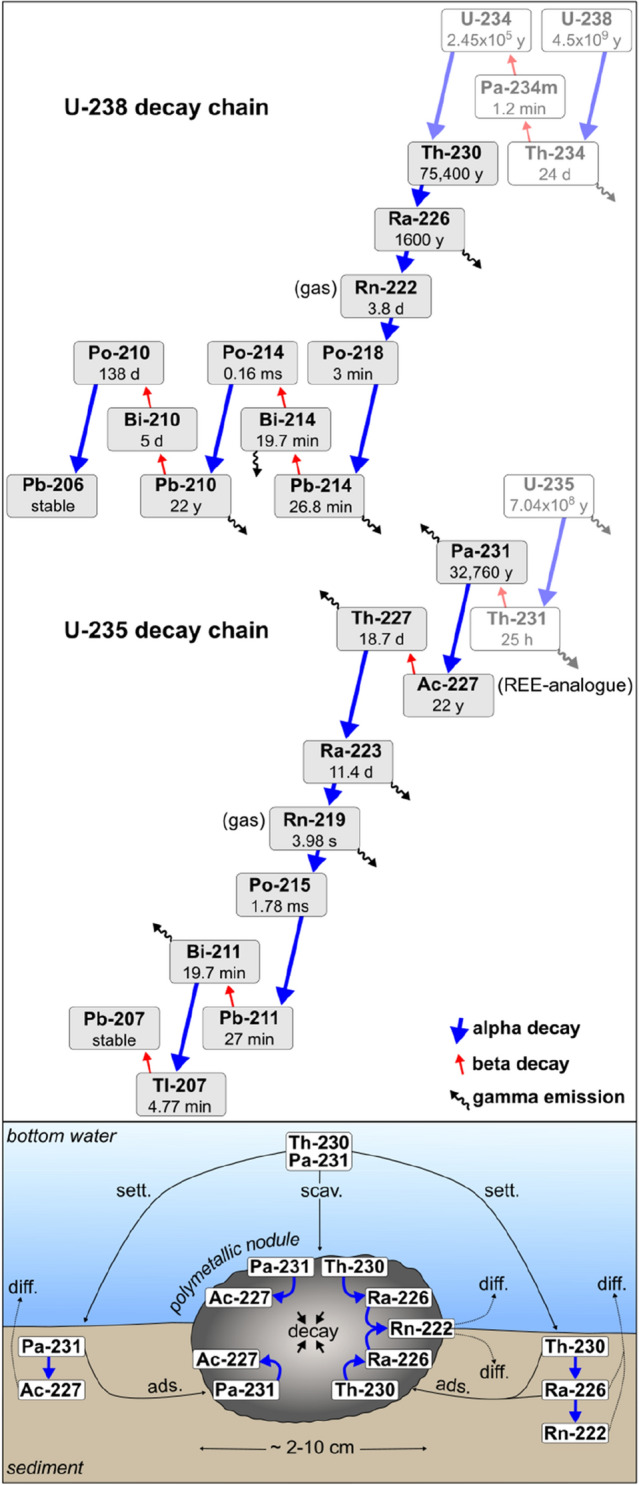
Visualization of the U-238 and U-235 decay chains and the scavenging, adsorption and settling processes involving uranium-series daughters in the slowly accumulating deep-sea environment in the presence of polymetallic nodules at the seafloor. *Scav* scavenging, *sett* settling, *diff* diffusion, *ads* adsorption. In the sediments of the NE Pacific Ocean, the activity concentrations of Th-230, Pa-231 and Ra-226 generally decrease with depth as they decay and maximum values in the upper 10 cm of the sediments reach up to 3.2 Bq g^−1^, 0.08 Bq g^−1^ and 1.5 Bq g^−1^, respectively^14,15^. Thus, activity concentrations in the topmost 10 cm of the sediments are at least one order of magnitude lower compared to polymetallic nodules but may still exceed NORM threshold values (cp. Table 1).

Naturally occurring radiation is responsible for the exposure of humans to a certain background radiation, which is usually not considered a significant health risk. NORM include all radioactive elements found in the environment and specifically denote materials where human activities have increased the potential for exposure (e.g., mining). In the context of NORM, the radionuclides of the U-238 and Th-232 decay series are particularly relevant. Due to the age of U-238 in deposits such as uranium ore, it can be assumed that the decay products Th-230 and Ra-226 are in secular equilibrium with U-238 (i.e., decay at the same rate). However, in partially open systems like the residues from ore processing or in nodules, isotopes need to be considered individually with respect to their radiation hazard as they are in disequilibrium with their parent isotopes (Fig. 1). Due to the enhanced exposure to ionizing radiation in industries that handle large quantities of NORM (e.g., coal, oil and gas industry, metal mining and smelting), regulations have been increasingly implemented in order to ensure occupational and public health and safety. For this purpose, governmental bodies have set activity concentrations for radioactive materials, at or below which some or all aspects of the regulatory control do not apply (i.e., exemption levels).

In comparison to the exemption levels, we present and discuss the activity concentrations of Th-230, Ra-226 and Pa-231 with focus on the nodule surface (see “Distribution of radioisotopes in polymetallic nodules”) as humans firstly and mostly come in contact with this part (see also abraded nodule dust in Fig. S2). Therefore, we first focus on maximum values found at the nodule surface as these levels are most relevant with regards to potential health risks and the exemption levels (Table 1) and because average values may lead to an underestimation of the risk from exposure to dust.

**Table 1:** Maximum activity concentrations from the nodule surface from new radioisotope data presented as part of this study (first row) and those found in published datasets (second row) in comparison to regulating exemption levels established by different authorities for selected radionuclides for unspecified materials and in naturally occurring radioactive materials (NORM).

Dataset/authority/guidelines	^230Th^ [Bq/g]	^226^Ra [Bq/g]	^231^Pa [Bq/g]	^222^Rn* [Bq/L]	Year of release	Material
Max. value in this study	7	9	0.55	–	–	Nodule surface
Max. value in literature	64^16^	21^16^	11^17^	–	–	Nodule surface
German Federal Office for radiation protection: German Radiation Ordinance (StrlSchV)^18^	0.1	0.01	0.01	–	2018	Solids and fluids
U.S. Environmental Protection Agency (EPA): 40 Code of Federal Regulations (CFR) Part 192*Health and Environmental Protection Standards for Uranium and Thorium Mill Tailings^19^	0.19	0.19	–	0.19	2018	Upper 15 cm of soil
UK Health and Safety Executive: Ionising Radiations Regulations (IRR17)^20^	0.1	0.01	0.01	0.3	2017	Any amount of material
Official Journal of the European Union: Basic safety standards on radiation protection 2013/59/Euratom^21^	1	1	1	–	2013	Solids
International Atomic Energy Agency (IAEA): Radiation Protection and Safety of Radiation Sources: International Basic Safety Standards^22^	1	1	1	0.3	2014	Large amounts of material (> 1 ton)
Health Canada: Canadian Guidelines for the Management of Naturally Occurring Radioactive Materials (NORM)^23^	10	0.3	–	0.2	2011	Solids

*Rn-222 exemption activity concentrations for air.

First row: max. values from new radioisotope data found in the outer ~ 2 mm of the nodules. Second row: published max. values found in the outer 0.2 mm (Th-230)^16^, 0.122–0.356 mm (Ra-226)^16^ and 7–12 mm (Pa-231)^17^ of the nodules.

National exemption levels vary among countries by two orders of magnitude (Table 1). International basic safety standards (BSS) have been set by the European Commission in the framework of the Council Directive 2013/59/EURATOM^21^ and by the International Atomic Energy Agency^22^, which stipulate exemption levels of 1 Bq g^−1^ for Th-230, Ra-226 and Pa-231 and 0.3 Bq L^−1^ for the inert radioactive gas Rn-222 for air (t_1/2_ = 3.82 days). This regulatory control includes the disposal of NORM wastes.

### Distribution of radioisotopes in polymetallic nodules

**Figure 2:**
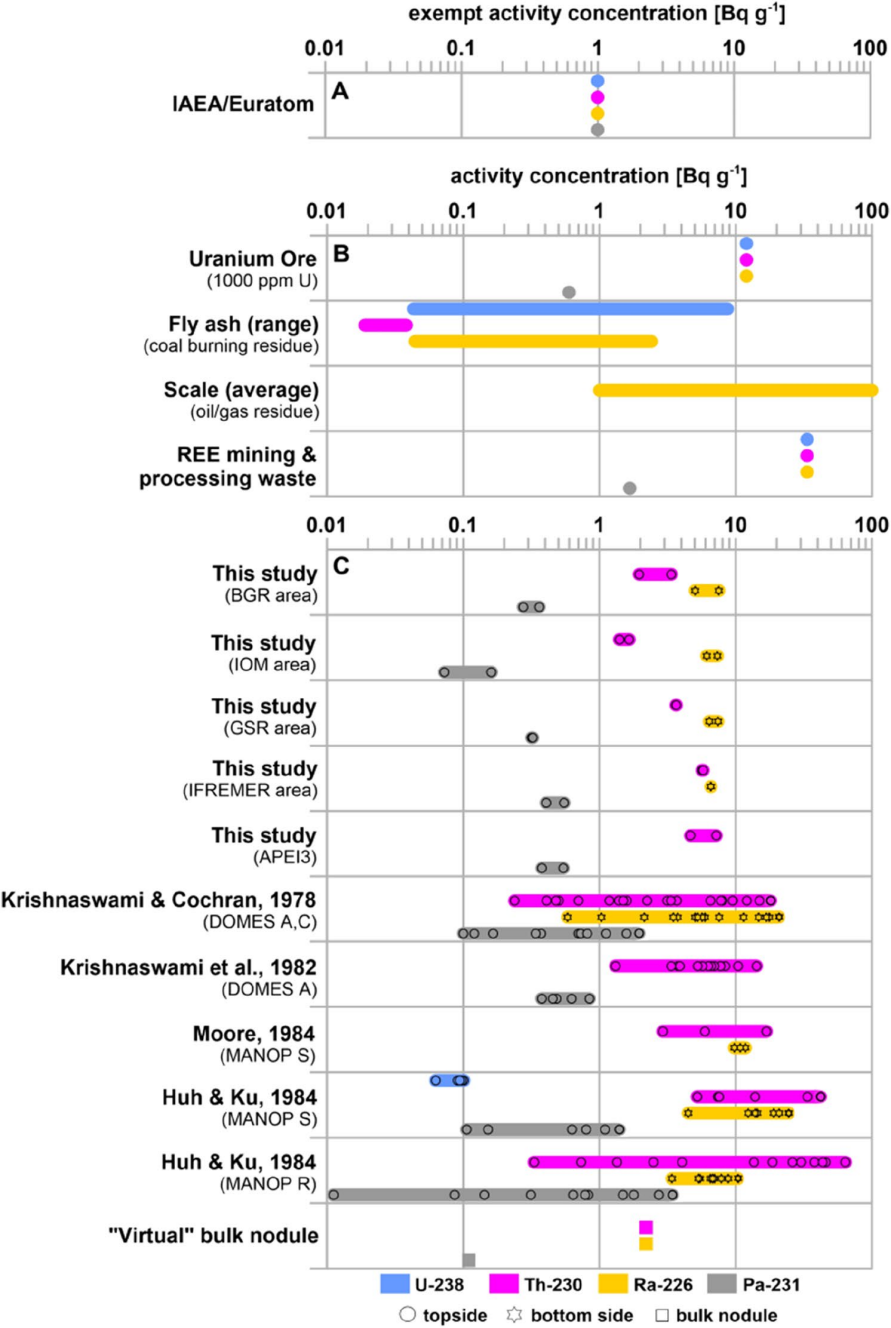
Overview of (**A**) international exemption levels, (**B**) activity concentrations of U-238, its daughters Th-230, Ra-226 and of Pa-231 in naturally occurring radioactive materials (NORM)^8,28^ and (**C**) new radioisotope data produced as part of this study for nodules from different exploration areas and published activity concentrations for the outer max. 2 mm-layer of nodules in the Pacific Ocean^16,24–26^. For more details on international exemption levels cp. Table 1. See extracted published datasets in Supplementary Table S2. Activity concentrations for the “virtual” bulk nodule were calculated under the assumption that the nodule is a spheroid (d = 50 mm; h = 30 mm) with Th-230 values between 0.1 and 10 Bq g^−1^, a half-value layer of 1 mm and a dry bulk density of 1.5 g cm^−3^ Refs.^29,30^, see “Supplementary Text: Web application setup” and web application (https://jevolz.shinyapps.io/Nodule_alpha_radiation/). The value for Pa-231 was set based on the natural U-238/U-235 ratio of ~ 20:1.

Our results for polymetallic nodules from five different exploration areas in the CCZ (Supplementary Figs. S1, S2, Supplementary Table S1) show an extreme variability between the interior and the outside of nodules, reaching maximum activity concentrations of 7 Bq g^−1^ for Th-230, 0.55 Bq g^−1^ for Pa-231 and 9 Bq g^−1^ for Ra-226 (Table 1, Fig. 2) at the surface. Activity concentrations for these radioisotopes in nodules from the CCZ are abundantly published and reach values of up to 64 Bq g^−1^ for Th-230, 11 Bq g^−1^ for Pa-231 and 21 Bq g^−1^ for Ra-226 at the surface of the nodules (Table 1, Supplementary Table S2)^16,17,24–27^. When comparing all of these activity concentrations determined for the nodules from different regions in the CCZ, it becomes clear that the Th-230, Ra-226 and Pa-231 concentrations vary between the nodules and are substantially different at the topside surface of the nodule (i.e., facing the water column) compared to the bottom surface, which is mostly embedded in the sediment (Figs. 1, 2). Th-230 and Pa-231 activity concentrations are generally higher at the topside surface than at the bottom surface of the nodule, while Ra-226 behaves inversely and mostly shows higher activity concentrations at the bottom surface^16,17^. As scavenging (topside) and adsorption (bottom side) are restricted to the surface of the polymetallic nodules, the activity concentrations of Th-230, Pa-231 and Ra-226 decrease rapidly into the interior of the nodule as they decay radioactively. Within the outer 1 mm-layer of the nodules, Th-230 activity concentrations are generally reduced by half (i.e., half-value layer)^16,17,24–27^.

Our measured activity values are all above specific NORM threshold levels (Table 1), which will be further elucidated and discussed in the following sections. In direct comparison with the uranium reference material RGU-1 (400 ppm U)^28^, our measurements of Th-230, Ra-226 and Pa-231 at the topside surface of the nodules even exceed the activity of the uranium ore sample (Supplementary Fig. S3). The historic studies on nodules from the CCZ reported values even up to one order of magnitude higher than our data (Fig. 2). These variations in surface nodule activity concentrations may partly be associated with different thicknesses and sampling techniques of the radioactive outer layer but also be a result of the observed natural variability in depositional conditions, biogeochemical processes and element fluxes in sediments of the CCZ^31,32^. Assuming that nodules follow the trend of Th-230 and Pa-231 in marine sediments^33,34^, one may thus also expect a lateral variability in the activity concentrations as a function of water depth and accumulation rates.

### Radiation risks during handling and processing of polymetallic nodules

The radioisotopes relevant during handling and processing of polymetallic nodules and deep-sea sediments mainly emit alpha radiation (i.e., alpha particles) during decay (Fig. 1). Alpha particles have a limited travel distance in air of a few centimeters and low penetration (i.e., they are stopped by paper). Therefore, conventional detectors for ionizing radiation, especially those with a thick end window to detect photon and beta radiation dose rates, may not always be suitable and alpha radiation can be easily underestimated. Most alpha particles do not penetrate the skin, and thus, external exposure to alpha radiation is not a major concern. However, alpha particles have high energies (MeV), and alpha emitters are considered particularly harmful if they enter the body through inhalation or ingestion. Therefore, in the following section, we successively discuss three main mechanisms that could lead to harmful alpha radiation exposure from nodules: (1) the inhalation or ingestion of highly radioactive dust from the surface of nodules, (2) the inhalation of radon gas emanating from nodules stored in an enclosed space and (3) the inhalation of nodules fines from entire nodules and the potential concentration of selected radionuclides during later nodule processing. These ways of exposure are most relevant in the context of deep-sea mining and subsequent metallurgical processing but are also important to be considered during scientific investigations.

### Health risks of polymetallic nodule mining

After polymetallic nodules are recovered from the seafloor, they are most likely crushed into smaller pieces (< 2 cm)^36^. Once they reach the mining platform (Supplementary Fig. S4), the nodule fragments are partially dried. Thus, their strength decreases as internal sediment (clay) layers dry out and lose their cohesive properties^35^. The dried nodule fragments then likely further disintegrate spontaneously due to attrition at the mining platform and in the bulk carrier during the transport to the shore (Supplementary Fig. S4). As a result, a fine fraction of nodule particles likely distributes in air, which can be inhaled or ingested by humans during the operation. The fine dust most likely consists of abraded material from the surface of the nodule fragments, where the activities of Th-230, Ra-226 and Pa-231 reach highest values (Fig. 2). Without safety precautions (e.g., respiratory mask with particle filter), some particles may only reach into the upper part of the lungs and can be exhaled. However, fine particles likely reach into the deep lungs and remain there. There, incorporated alpha emitters can damage cells, and thus, impose a great radiological hazard for the human body, as reflected in very low exemption levels of 0.01 Bq g^−1^ in some national radiation safety regulations (e.g., Germany^18^ and UK^20^; Table 1). Furthermore, residuals from the mining slurry (mixture of seawater, sediment and nodule fines) are released back into the ocean as a discharge plume. Of the total mass of the produced nodules, < 7% abraded nodule fragments and < 20% of entrained sediments may be discharged^36–39^. Although the discharge plume is expected to be released below the photic zone and the oxygen minimum zone^39^, radioactive particles from the plume can be ingested by marine organisms, and thus, may enter the food chain and ultimately be ingested by humans^40^.

**Figure 3:**
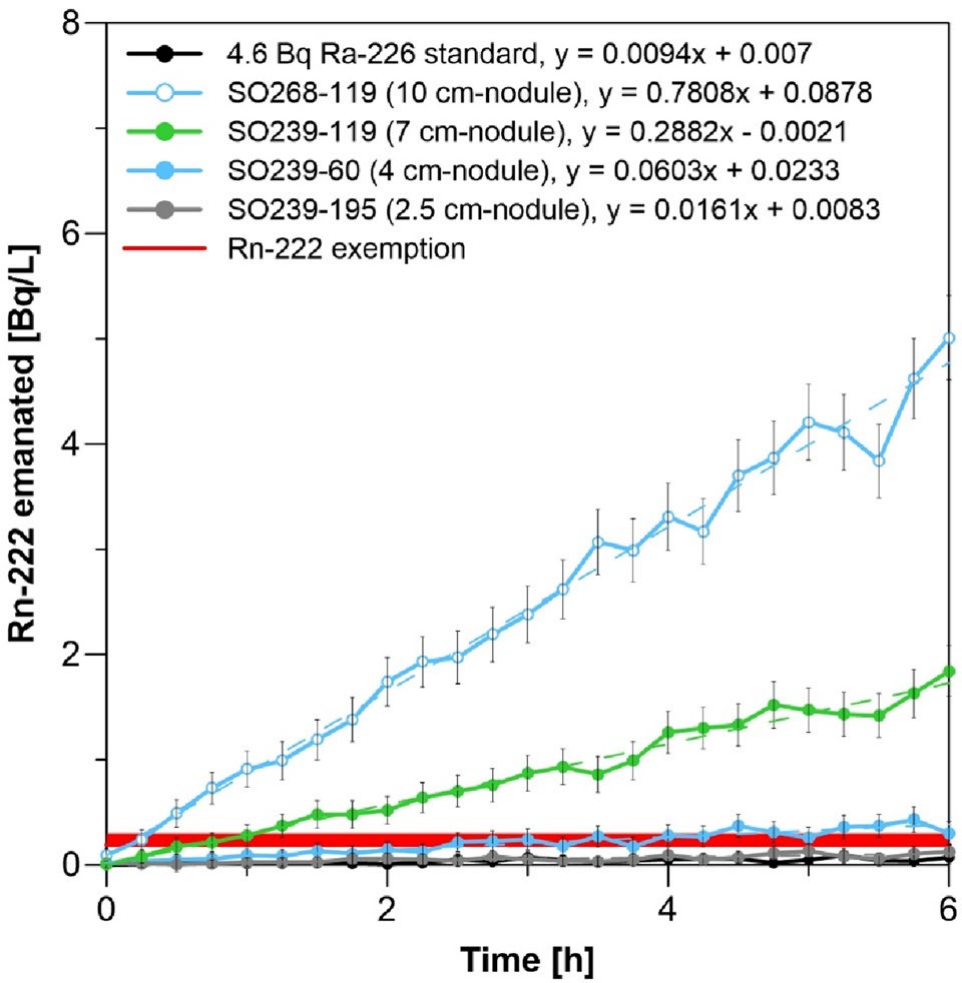
Radon emanation of four different nodules with different sizes and from different exploration areas (cp. Table S1, Figs. S1, S2). The nodules were placed into a closed sample glass chamber (total system volume 2.02 L) and Rn-222 emanation was continuously measured for 6 h. The error bars indicate the calculated 2-sigma uncertainty. For comparison, 4.6 Bq of a Ra-226 standard is shown. In addition, the range of Rn-222 exemption levels (cp. Table 1) is indicated.

Nodules are a source for Rn-222 from the decay of Ra-226^41^, which becomes a health issue when nodules are stored in bulk in enclosed, unventilated spaces. Once inhaled by humans, most Rn-222 is immediately exhaled but its relatively short-lived (< 3 min) progeny (polonium-218, polonium-214) may deposit in the lungs emitting alpha radiation^42^. If stored in an enclosed space, Rn-222 activity concentrations increase with time. Our results show that a single 100 mm-diameter nodule produces ~ 5 Bq L^−1^ after ~ 6 h of storage in a 2-L air volume (Fig. 3; corresponding to a production of ~ 10 Bq). For comparison, average background Rn-222 activity concentrations reach up to 0.01 Bq L^−1^ in the atmosphere and 0.05 Bq L^−1^ indoors^43^.

Generally, the emanation of Rn-222 positively correlates with the nodule size with specific release rates of 0.07–0.101 Bq cm^−2^ day^−1^ (see “Supplementary Text: Radon accumulation in dependency of the nodule size”). The produced Rn-222 levels in air may exceed current Rn-222 exemption levels of 0.2–0.3 Bq L^−1^ (Table 1) depending on air volume, nodule size and storage duration (Fig. 3). For example, a single 100 mm-diameter nodule stored in an unventilated small room (10 m^3^) will produce Rn-222 concentrations of 0.015 Bq L^−1^, which do not surpass exemption levels and these concentrations are much lower if the room is ventilated. Projected on large-scale mining operations, the emanation of Rn-222 becomes a serious radiation risk if the nodules are stored in enclosed spaces such as in bulk carriers (Supplementary Fig. S4). As the bulk carriers will most likely load mixtures of different nodule sizes, it is difficult to assess how much Rn-222 may be produced during transport. For an approximation of the Rn-222 emanation in bulk carriers, we assume that the transport of nodules from the CCZ to the nearest port in Mexico will at least take 3 days^44^ and that with a deadweight of 50,000 tons and an average dry nodule bulk density of 1.5 g cm^−3^ Refs.^29,30^, ~ 33 million dm^3^ of nodules are transported. Based on our results on the Rn-222 emanation of single nodules (Fig. 3), the approximately 22 million dm^3^ of air in the pore space in a shipload of nodules will most certainly have Rn-222 concentrations that exceed current exemption levels (0.2–0.3 Bq L^−1^; Table 1) by orders of magnitude (200–1000 Bq L^−1^; Supplementary Table S4). Unless the bulk carriers are constantly ventilated, human protection from inhaling this radioactive noble gas in air seems impossible.

As shown earlier, alpha emitters are distributed heterogeneously within the nodule (Fig. 2), and therefore, the health risk from inhaling and ingesting nodule dust highly depends on the exposure type. For the metallurgical extraction of metals on land (Supplementary Fig. S4), the entire nodule (fragments) has/have to be processed, which makes it necessary to consider the total alpha radiation. As reflected by the highest activity concentrations, Th-230 is most effectively scavenged onto the nodule surface compared to other radioisotopes (Fig. 2)^16,17,24–27^. While daughters of Th-230 contribute to the alpha radiation exposure, we use the example of Th-230 to calculate the approximate bulk nodule alpha radiation. We have used a basic numerical approach (see “Supplementary Text: Web application setup”) to calculate the bulk nodule Th-230 inventory based on radioisotope data (Fig. 2) and created a web application for public use (https://jevolz.shinyapps.io/Nodule_alpha_radiation/). Assuming that a “virtual” nodule is a spheroid with a 50-mm equatorial diameter (size range 20–80 mm^45^) and a height of 30 mm, assumed Th-230 values between 0.1 (inside) and 10 Bq g^−1^ (surface), a half-value layer of 1 mm and 1.5 g cm^−3^ for the dry bulk density^29,30^, we find a bulk inventory of the “virtual” nodule of ~ 2.2 Bq g^−1^. For smaller nodules, the bulk Th-230 inventory generally increases as the nodule mass decreases. According to our calculation, only large nodules (d > 98 mm; h > 88 mm) would have bulk Th-230 inventories below 1 Bq g^−1^, which still falls within the range of current exemption levels of 0.1–10 Bq g^−1^ (Table 1) and particles from their surface would have activities considerably exceeding exemption limits. Thus, if entire nodules are crushed and ground, fine particles are released, which have lower activity concentrations than at the surface but they still can pose health risks if handled without safety precautions.

To our knowledge, the fate of the abundant actinides during the metal extraction from nodules on land (Supplementary Fig. S4) has not yet been studied and we use the example of actinium-227 (Ac-227) in order to outline the behavior in such a procedure. Ac-227 (t_1/2_ = 21.77 years) is a decay product of Pa-231 (Fig. 1), and due to the relatively short half-life of Ac-227 compared to Pa-231 (t_1/2_ = 32,760 years), the activities of both radioisotopes are assumed to be very similar, in the order of 0.1–10 Bq g^−1^ on the outside of nodules (Fig. 2). The chemical properties of actinium closely resemble those of lanthanum^46^ and therefore Ac-227 is concentrated together with the rare earth elements (REE)^47^. If approximately 1 mg g^−1^ of total REE are present in nodules^2^, and Ac-227 at an average of ~ 0.1 Bq g^−1^, a purified REE fraction from nodules would contain 100 Bq g^−1^ Ac-227, which exceeds exemption levels by up to 1000 (Table 1). Consequently, the REE extract from nodules, which is supposed to be used in the production of high technology may carry substantial radioactivity.

In summary, our results demonstrate that the collection, transport, storage and processing of polymetallic nodules (and of deep-sea sediments) should be considered under radiation safety aspects in future activities. The new radioisotope dataset presented in this study validates the alarmingly high activity concentrations published in historic studies and vice versa. These values exceed current national and international exemption levels by up to three orders of magnitude. This exceedance of the exemption limits has, to our knowledge, not yet been taken into consideration for the legal framework of mining operations. We show that the anticipated nodule mining procedure threatens the occupational and public health through the likely inhalation or ingestion of radioactive nodule dust and of emanating Rn-222 gas, potentially through the uptake of alpha emitters via the marine food chain and possibly their concentration during metal extraction on land. In due consideration of the activity concentrations above current exemption levels for several alpha emitters in polymetallic nodules and with associated measures to avoid/reduce health risks, costs for deep-sea mining are expected to rise, questioning its viability.

## Methods

### Sample location, sampling and processing

In the framework of the MiningImpact2 project, the RV SONNE cruises SO239 in 2015^48^ and SO268 in 2019^49^ took place in the Clarion-Clipperton Zone (CCZ) in the NE Pacific Ocean. During these cruises, different exploration areas for polymetallic nodules were visited, including the German BGR (Bundesanstalt für Geowissenschaften und Rohstoffe/Federal Institute for Geosciences and Natural Resources) area, the IOM (Interoceanmetal) Joint Organization area, the Belgian GSR (Global Sea Mineral Resources NV) area, the French IFREMER (Institut français de recherche pour l'exploitation de la mer) area and the Area of Particular Environmental Interest (APEI) No. 3, which is excluded from mining activities (Supplementary Fig. S1, Supplementary Table S1). During cruise SO239, a boxcorer was used for the collection of seafloor nodules from an area of 0.25 m^2^ Ref.^48^. The collected polymetallic nodules were cleaned of adhered sediments and stored in plastic bags at room temperature until further analysis. During cruise SO268, a small dredge was used in the German BGR area, which collected polymetallic nodules from the seafloor^49^. One of the largest nodule was cleaned of adhered sediments, wrapped in tissues, which were soaked with seawater and stored at 4 °C until further analysis.

For this study, intact polymetallic nodules with different sizes were selected from different exploration areas (Supplementary Fig. S2, Supplementary Table S1). For the measurement of Radon emanation, intact nodules from four stations were chosen, which had different sizes (Supplementary Fig. S2). Hereafter, samples were taken from the topside surface (s), the bottom surface (b) and from the interior (i) of the nodules sampled during cruise SO239, using suitable safety precautions regarding inhalation. These samples were freeze-dried and homogenized for the analysis using gamma spectrometry.

### Radon emanation

Radon emanation was measured by enclosing nodules of various size (n = 4) separately in a glass chamber while continuously circulating the gas in the system through a Rad7 device (Durridge Inc, calibrated by manufacturer). This system is designed to measure specifically radon. Results were evaluated with the accompanying Capture^®^ software. For a description of the counting system, we refer to Xu et al.^50^. Our closed sample glass chamber differs from their setup as it has only 2.02 L volume (Rad7 volume: 0.8 L). As a comparison, 4.6 Bq of a Ra-226 standard in dilute hydrochloric acid solution were neutralized with dilute sodium hydroxide and placed in the glass chamber after adding it onto a manganese-coated acrylic fiber (expected 0.84 Bq day^−1^ at full emanation).

Absolute activities of Rn-222 here should be taken with a grain of salt as they represent minimum estimates. One reason is that the recoil efficiencies of the nodules are not known. For a somewhat comparable setup, as used in the RaDeCC^51^, recoil and consequently Rn-222 emanation from manganese-coated fiber has been shown to depend on the presence of water and possibly on the carrier gas. In addition, one has to consider the fact that the Rad7 system is not designed for keeping a completely closed gas circuit, which leads to a faster loss in the system than just expected by decay^50^. Mathematically, diffusive gas loss is proportional to the activity and has the effect of increasing the apparent decay constant. The measured emanation from the standard was only ~ 69% of the value expected when all Rn-222 was released. Using the Ra-226 standard, our diffusive gas loss term was estimated to be 0.43*day^−1^. In a closed Rad7, the effect of this loss increases proportional to Rn-222 activity, so it is lowest at the beginning of the nodule incubation. With a gas loss of 0.43*day^−1^, we find that after six hours of counting, the observed activity is approximately 7% lower than it would be without diffusive gas loss. Therefore, we only report the first six hours of counting, in which the increase by production from Ra-226 dominates over possible loss terms, and we consider the reported radon activities a minimum value. The slope of the radon increase over time is still proportional to radon emanation from the samples^52^, and a direct comparison of the nodules of different size is therefore fully appropriate.

### Gamma spectrometry

Nodule samples from five stations, including similar replicates (Supplementary Fig. S2) were taken from different parts of the nodules (topside surface (s), bottom surface (b) and interior (i). The dried samples were ground to a fine powder, using appropriate safety measures consisting in a particle-filtering face mask and white surfaces to prevent spreading of particles. Amounts of 0.5–1 g were filled into polypropylene tubes (Sarstedt^®^ 11.5 mm diameter, 60 mm length) to a filling height of approximately 1 cm and the exact mass was noted. The tubes were additionally sealed with parafilm^®^ below the lid to prevent the loss of radon. Tightness of this sealing type was previously tested by measuring radon-containing air and monitoring decay over several Rn-222 half-lives, and only minor gas losses were observed. Together with the samples, diluted uranium ore standard RGU-1^28^ and uranium ore standard UREM-11^53^ were prepared in similar amounts and geometry. RGU-1 served as a calibration, and UREM-11 was used as a reference material to verify the results. The gamma radiation from the samples was measured in a Canberra well-type HPGe detector (126 cm^3^ volume, 1.35 keV resolution at 122 keV). Spectra were analyzed using the ScientissiMe and InterWinner software. We evaluated the emission lines at 46 keV (Pb-210), 63 keV (Th-234 as an indirect measure of U-234), 67 keV (Th-230), 186 keV (Ra-226 in the absence of a U-235 interference), 295 keV (Rn-222 as an indirect measure of Ra-226) and 402 keV (Rn-219 as an indirect measure of Pa-231, with some uncertainty on the intermediate Pa-231/Ac-227 equilibrium). The verification of ^231^Pa and ^230^Th via gamma counting was difficult because UREM-11 is much lower in both isotopes, leading to high uncertainties in this reference material.

## Supplementary Information

Supplementary Information.

## Data Availability

The radioisotope datasets produced in the framework of this study are available at the PANGAEA database (doi: https://doi.org/10.1594/PANGAEA.951145; doi: https://doi.org/10.1594/PANGAEA.951148).
