# Supplementary material for: Alpha radiation from polymetallic nodules and potential health risks from deep-sea mining
Source: Sci Rep. 2023 May 17;13:7985. doi: 10.1038/s41598-023-33971-w (PMC10192382; doi:10.1038/s41598-023-33971-w)
Supplement: Supplementary file 1 — Supplementary Information. [file 41598_2023_33971_MOESM1_ESM.pdf]

Supplementary information

Alpha radiation from polymetallic nodules and potential health risks from deep-sea mining

Jessica B. Volz[1,*], Walter Geibert[1], Dennis Köhler[1], Michiel M. Rutgers van der Loeff[1] and Sabine Kasten[1,2,3]

[1] Alfred Wegener Institute Helmholtz Centre for Polar and Marine Research, Am Handelshafen 12, 27570 Bremerhaven, Germany

[2] University of Bremen, Faculty of Geosciences, Klagenfurter Strasse 4, 28359 Bremen, Germany

[3] MARUM – Center for Marine Environmental Sciences, Leobener Strasse 8, 28359 Bremen, Germany

*Corresponding author:

Tel: +49 471 4831 1842

Email: Jessica.volz@awi.de

Supplementary Text: Web application setup

For the setup of the web application for the calculation of the bulk nodule Th-230 activity for public use (https://jevolz.shinyapps.io/Nodule_alpha_radiation/), we used the shiny package for R Studio. The calculation is based on the assumption that the nodule is spheroid-shaped (Supplementary Fig. S5). The total volume of the nodule (V_{nodule}) is dependent on the nodule height (h) and equatorial diameter (d) chosen by the user of the web application:

$$V_{nodule} = \frac{1}{6} * \pi * h * d^2 \quad (1)$$

The surface area of the nodule (S_{nodule}), which is assumed to be an oblate spheroid (h<d), is given by:

$$S_{nodule} = \frac{1}{2}\pi d^2 + \pi \frac{h^2}{4e} ln\left(\frac{1+e}{1-e}\right) \quad (2)$$

where e is the eccentricity:

$$e = \sqrt{1 - \frac{h^2}{d^2}} \quad (3)$$

The Th-230 activity is highest at the surface of the nodule with the activity A_0 (Bq/g) and decreases exponentially with depth z into the nodule with the half-value depth HVD and the decay constant $\beta = \frac{\ln(2)}{HVD}$ to a background activity A_{int} in the interior of the nodule. Assuming that the Th-230-rich layer is thin in comparison with the nodule size (HVD<<d,h), the Th-230 distribution is treated as 1-dimensional and the integrated excess activity per unit of surface area $I_{exc,sa}$ of the nodule is:

$$I_{exc,sa} = \int_0^\infty (A_0 - A_{int}) DBD\, e^{-\beta z} dz = (A_0 - A_{int}) DBD \frac{HVD}{\ln(2)} \quad (4)$$

where DBD is dry bulk density. With the same assumption of a thin Th-230-rich layer, the total excess Th-230 inventory ($I_{exc,total}$) in the nodule is approximated as:

$$I_{exc,total} = I_{exc,sa} * S_{nodule} \quad (5)$$

The total Th-230 inventory of the nodule is the sum of the background (I_{bg}) and the excess activity:

$$I_{nodule} = I_{exc,total} + I_{bg} = I_{exc,sa} * S_{nodule} + A_{int} * DBD * V_{nodule} \quad (6)$$

And the bulk nodule average activity (A_{bulk}) of Th-230 is:

$$A_{bulk} = \frac{I_{nodule}}{V_{nodule} * DBD} = \frac{I_{exc,sa} * S_{nodule}}{V_{nodule} * DBD} + A_{int} \tag{7}$$

Supplementary Text: Radon accumulation in dependency of the nodule size

From the results of the Rn-222 emanation experiments shown in Fig. 3, we determined a linear Rn-222 concentration increase rate (a) [Bq L^{-1} d^{-1}] in the chamber. In the next step, we calculated the Rn-222 emanation rate (E) [Bq cm^{-2} d^{-1}] from each nodule (Table S3) using the surface of the nodule (S_{nodule}; equation (2)) and the total gas volume in the experimental setup (V_{tot}) of 2.02 L, corrected for the volume of the nodule (V_{nodule}; equation (1)):

$$E = \frac{a * (V_{tot} - V_{nodule})}{S_{nodule}} \tag{8}$$

If nodules are stored dry with a packing factor F and porosity φ ($\varphi = 1 - F$), Rn-222 will accumulate in the pore space with the decay constant (λ) over time (t) with:

$$A = \frac{E S_{nodule}}{\lambda V_{nodule}} \frac{(1-\varphi)}{\varphi} (1 - e^{-\lambda t}) \tag{9}$$

With a 50,000 ton cargo of dry nodules, Rn-222 will accumulate in the pore space of:

$$50000 * \frac{\varphi}{DBD(1-\varphi)} \text{ m}^3 \tag{10}$$

or about 22,000 m^3 for an assumed packing factor F of 0.6 and a nodule dry bulk density of 1.5 g cm^{-3}. We performed the calculation of the Rn-222 activity (A) accumulated in this volume of air for different nodule sizes ($d = 20\text{–}80$ mm) representative for the size range of nodules found in the CCZ[45] (Supplementary Table S4). With regards to mining operations, we assume that the Rn-222 emanation does not greatly change due to crushing of nodules. Depending on the initial nodule size and the surface emanation rate, different Rn-222 concentrations are reached after one half-life of 3.8 days (Supplementary Table S4), which represents roughly the transit time to the nearest harbour.

Supplementary Tables

Table S1: Detailed station information for the study sites of the presented study in the different exploration areas for polymetallic nodules. The geographical position and water depth for SO268-119 represent the start of the dredge track.

Exploration area	Cruise	Station	Gear	Lat N	Long W	Water depth [m]
German BGR	SO268	119	Dredge	11.863°	117.015°	4122.2
German BGR	SO239	60	Boxcorer	11.807°	117.55°	4324.5
IOM Joint Org.	SO239	88	Boxcorer	11.079°	119.659°	4432.9
Belgian GSR	SO239	119	Boxcorer	13.859°	123.253°	4516.2
French IFREMER	SO239	180	Boxcorer	14.042°	130.136°	4936.4
APEI3	SO239	195	Boxcorer	18.796°	128.362°	4833.4

Table S2: Summary of extracted published radioisotope datasets from historical studies performed with polymetallic nodules from the Clarion-Clipperton Zone (CCZ) in the NE Pacific Ocean as part of the Deep Ocean Mining Environmental Study (DOMES), Manganese Nodule Project (MANOP) and the Korea Deep Ocean Study (KODOS).

Site	Sample ID	Lat N	Long W	Water depth [m]	Nodule side	Depth min [mm]	Depth max [mm]	U-238 [Bq/g]	Th-230 [Bq/g]	Ra-226 [Bq/g]	Pa-231 [Bq/g]	Th-232 [Bq/g]	Data source
DOMES A	RP8OC75-47-16-1-1T1	09.04°	151.19°	4948	top	0	0.245	n.a.	7.97	2.88	0.70	0.11	Krishnaswami and Cochran (1978)[24]
DOMES A	RP8OC75-47-16-1-1T2	09.04°	151.19°	4948	top	0.245	0.53	n.a.	3.70	2.57	0.17	0.10	
DOMES A	RP8OC75-47-16-1-1T3	09.04°	151.19°	4948	top	0.53	0.855	n.a.	2.23	2.23	n.a.	0.09	
DOMES A	RP8OC75-47-16-1-1T4	09.04°	151.19°	4948	top	0.855	1.16	n.a.	1.19	1.29	n.a.	0.08	
DOMES A	RP8OC75-47-16-1-1T5	09.04°	151.19°	4948	top	1.16	1.48	n.a.	0.70	0.77	n.a.	0.09	
DOMES A	RP8OC75-47-16-1-1T6	09.04°	151.19°	4948	top	1.48	1.84	n.a.	0.24	0.38	n.a.	0.06	
DOMES A	RP8OC75-47-16-1-1T7	09.04°	151.19°	4948	top	1.84	2.28	n.a.	0.24	0.30	n.a.	0.07	
DOMES A	RP8OC75-47-16-1-1T8	09.04°	151.19°	4948	top	15	15.2	n.a.	0.24	0.24	n.a.	0.07	
DOMES A	RP8OC75-47-16-1-1B1	09.04°	151.19°	4948	bottom	0	0.235	n.a.	1.61	14.80	0.11	0.04	
DOMES A	RP8OC75-47-16-1-1B2	09.04°	151.19°	4948	bottom	0.235	0.36	n.a.	2.27	11.42	n.a.	0.12	
DOMES A	RP8OC75-47-16-1-1B3	09.04°	151.19°	4948	bottom	0.36	0.7	n.a.	1.82	7.58	n.a.	0.18	
DOMES A	RP8OC75-47-16-1-1B4	09.04°	151.19°	4948	bottom	0.7	0.85	n.a.	1.09	5.05	n.a.	0.13	
DOMES A	RP8OC75-47-16-1-1B5	09.04°	151.19°	4948	bottom	0.85	1	n.a.	0.61	3.75	n.a.	0.13	
DOMES A	RP8OC75-47-16-1-2T1	09.04°	151.19°	4948	top	0	0.106	n.a.	14.98	2.73	1.59	0.13	
DOMES A	RP8OC75-47-16-1-2T2	09.04°	151.19°	4948	top	0.106	0.232	n.a.	9.55	2.68	0.73	0.14	
DOMES A	RP8OC75-47-16-1-2T3	09.04°	151.19°	4948	top	0.232	0.352	n.a.	7.80	3.30	0.37	0.16	
DOMES A	RP8OC75-47-16-1-2T4	09.04°	151.19°	4948	top	0.352	0.535	n.a.	3.15	2.07	0.10	0.13	
DOMES A	RP8OC75-47-16-1-2T5	09.04°	151.19°	4948	top	0.535	0.713	n.a.	1.39	1.41	n.a.	0.12	
DOMES A	RP8OC75-47-16-1-2T6	09.04°	151.19°	4948	top	0.713	0.939	n.a.	0.51	0.94	n.a.	0.12	
DOMES A	RP8OC75-47-16-1-2T7	09.04°	151.19°	4948	top	0.939	1.2	n.a.	0.48	0.75	n.a.	0.11	
DOMES A	RP8OC75-47-16-1-2T8	09.04°	151.19°	4948	top	1.21	1.4	n.a.	0.41	0.72	n.a.	0.13	
DOMES A	RP8OC75-47-16-1-2B1	09.04°	151.19°	4948	bottom	0	0.179	n.a.	1.59	17.58	0.11	0.06	
DOMES A	RP8OC75-47-16-1-2B2	09.04°	151.19°	4948	bottom	0.179	0.264	n.a.	1.72	16.83	0.09	0.13	
DOMES A	RP8OC75-47-16-1-2B3	09.04°	151.19°	4948	bottom	0.264	0.515	n.a.	0.96	0.00	n.a.	0.13	
DOMES A	RP8OC75-47-16-1-2B4	09.04°	151.19°	4948	bottom	0.515	1.03	n.a.	0.46	5.93	n.a.	0.13	

Site	Sample ID	Lat N	Long W	Water depth [m]	Nodule side	Depth min [mm]	Depth max [mm]	U-238 [Bq/g]	Th-230 [Bq/g]	Ra-226 [Bq/g]	Pa-231 [Bq/g]	Th-232 [Bq/g]	Data source
DOMES A	RP8OC75-47-16-1-2B5	09.04°	151.19°	4948	bottom	1.22	1.4	n.a.	0.23	3.48	n.a.	0.06	Krishnaswami et al. (1982)[25]
DOMES A	RP8OC75-47-16-1A1	09.04°	151.19°	4948	top	0	0.073	n.a.	14.20	n.a.	n.a.	0.16	
DOMES A	RP8OC75-47-16-1A2	09.04°	151.19°	4948	top	0.073	0.107	n.a.	10.40	n.a.	n.a.	0.16	
DOMES A	RP8OC75-47-16-1A3	09.04°	151.19°	4948	top	0.107	0.162	n.a.	8.38	n.a.	0.84	0.12	
DOMES A	RP8OC75-47-16-1A4	09.04°	151.19°	4948	top	0.162	0.224	n.a.	7.73	n.a.	0.63	0.14	
DOMES A	RP8OC75-47-16-1A5	09.04°	151.19°	4948	top	0.224	0.256	n.a.	7.77	n.a.	n.a.	0.15	
DOMES A	RP8OC75-47-16-1A6	09.04°	151.19°	4948	top	0.256	0.306	n.a.	6.97	n.a.	0.48	0.14	
DOMES A	RP8OC75-47-16-1A7	09.04°	151.19°	4948	top	0.306	0.358	n.a.	6.62	n.a.	0.45	0.12	
DOMES A	RP8OC75-47-16-1A8	09.04°	151.19°	4948	top	0.358	0.43	n.a.	6.28	n.a.	0.38	0.13	
DOMES A	RP8OC75-47-16-1A9	09.04°	151.19°	4948	top	0.43	0.482	n.a.	5.68	n.a.	n.a.	0.14	
DOMES A	RP8OC75-47-16-1A10	09.04°	151.19°	4948	top	0.482	0.544	n.a.	5.27	n.a.	n.a.	0.13	
DOMES A	RP8OC75-47-16-1A11	09.04°	151.19°	4948	top	0.544	0.637	n.a.	3.77	n.a.	n.a.	0.12	
DOMES A	RP8OC75-47-16-1A12	09.04°	151.19°	4948	top	0.637	0.701	n.a.	3.35	n.a.	n.a.	0.13	
DOMES A	RP8OC75-47-16-1A13	09.04°	151.19°	4948	top	0.701	0.756	n.a.	3.88	n.a.	n.a.	0.20	
DOMES A	RP8OC75-47-16-1A14	09.04°	151.19°	4948	top	0.756	1.76	n.a.	1.32	n.a.	n.a.	0.12	
DOMES B	RP8OC75-55-56-1-KT1	11.81°	137.41°	4800	top	0.05		n.a.	7.78	n.a.	n.a.	n.a.	Kadko and Burckle (1980)[54]
DOMES B	RP8OC75-55-56-1-KT2	11.81°	137.41°	4800	top	0.15		n.a.	0.35	n.a.	n.a.	n.a.	
DOMES B	RP8OC75-55-56-1-KT3	11.81°	137.41°	4800	top	0.25		n.a.	0.04	n.a.	n.a.	n.a.	
DOMES B	RP8OC75-55-56-1-KB1	11.81°	137.41°	4800	bottom	0.05		n.a.	0.19	n.a.	n.a.	n.a.	
DOMES B	RP8OC75-55-56-1-KB2	11.81°	137.41°	4800	bottom	0.15		n.a.	0.03	n.a.	n.a.	n.a.	
DOMES C	RP8OC75-57-58-1-1T1	15.33°	125.91°	4551	top	0	0.038	n.a.	12.08	2.42	1.12	0.11	Krishnaswami and Cochran (1978)[24]
DOMES C	RP8OC75-57-58-1-1T2	15.33°	125.91°	4551	top	0.038	0.129	n.a.	9.50	3.18	0.81	0.13	
DOMES C	RP8OC75-57-58-1-1T3	15.33°	125.91°	4551	top	0.129	0.238	n.a.	6.50	3.00	0.34	0.16	
DOMES C	RP8OC75-57-58-1-1T4	15.33°	125.91°	4551	top	0.238	0.347	n.a.	3.33	2.58	0.12	0.13	
DOMES C	RP8OC75-57-58-1-1T5	15.33°	125.91°	4551	top	0.347	0.447	n.a.	1.59	1.97	n.a.	0.11	
DOMES C	RP8OC75-57-58-1-1T6	15.33°	125.91°	4551	top	0.447	0.576	n.a.	1.49	1.75	n.a.	0.12	
DOMES C	RP8OC75-57-58-1-1T7	15.33°	125.91°	4551	top	0.714	0.845	n.a.	0.41	1.06	n.a.	0.07	
DOMES C	RP8OC75-57-58-1-1B1	15.33°	125.91°	4551	bottom	0	0.42	n.a.	0.66	5.67	0.06	0.02	

Site	Sample ID	Lat N	Long W	Water depth [m]	Nodule side	Depth min [mm]	Depth max [mm]	U-238 [Bq/g]	Th-230 [Bq/g]	Ra-226 [Bq/g]	Pa-231 [Bq/g]	Th-232 [Bq/g]	Data source
DOMES C	RP8OC75-57-58-1-1B2	15.33°	125.91°	4551	bottom	0.42	0.91	n.a.	n.a.	5.27	n.a.	n.a.	
DOMES C	RP8OC75-57-58-1-1B3	15.33°	125.91°	4551	bottom	0.91	1.13	n.a.	0.08	2.13	n.a.	0.03	
DOMES C	RP8OC75-57-58-1-1B4	15.33°	125.91°	4551	bottom	1.13	1.35	n.a.	0.09	1.03	n.a.	0.04	
DOMES C	RP8OC75-57-58-1-1B5	15.33°	125.91°	4551	bottom	1.35	1.56	n.a.	0.08	n.a.	n.a.	0.04	
DOMES C	RP8OC75-57-58-1-1B6	15.33°	125.91°	4551	bottom	1.56	1.75	n.a.	n.a.	0.58	n.a.	n.a.	
MANOP S	Mn7601-20-2	11.07°	140.05°	4722	top	0	3	1.23	12.08	3.50	8.50	2.18	
MANOP S	Mn7601-20-2	11.07°	140.05°	4722	top	3	7	1.02	4.12	2.47	2.10	1.57	
MANOP S	Mn7601-20-2	11.07°	140.05°	4722	top	7	12	0.97	2.03	10.13	10.68	1.38	
MANOP S	Mn7601-20-2	11.07°	140.05°	4722	top	12	13	0.73	2.65	1.00	3.18	2.13	Moore et al. (1981)[17]
MANOP S	Mn7601-20-2	11.07°	140.05°	4722	top	13	15	0.70	9.60	9.60	1.72	1.13	
MANOP S	Mn7601-20-2	11.07°	140.05°	4722	top	15	17	0.63	5.75	5.75	0.57	0.95	
MANOP S	Mn7601-20-2	11.07°	140.05°	4722	top	22	28	0.50	1.65	1.65	0.35	0.77	
MANOP S	K7905-54BC-1-M1	11.02°	140.09°	4905	top	0	1	n.a.	5.95	3.27	n.a.	0.06	
MANOP S	K7905-62BC-1-M1	11.03°	140.10°	4906	top	0	2.1	n.a.	5.47	8.78	n.a.	0.07	
MANOP S	K7905-66BC-1-M1	11.02°	140.10°	4906	top	0	1.4	n.a.	16.77	2.28	n.a.	0.22	
MANOP S	K7905-75BC-1-M1	11.02°	140.09°	4913	top	0	3.5	n.a.	4.73	4.93	n.a.	0.07	
MANOP S	K7905-91BC-1-M1	11.02°	140.10°	4908	top	0	4.2	n.a.	5.73	3.57	n.a.	0.08	
MANOP S	K7905-101BC-1-M1	11.02°	140.08°	4924	top	0	1.5	n.a.	2.92	2.55	n.a.	0.04	
MANOP S	K7905-104BC-1-M1	11.02°	140.09°	4914	top	0	2.7	n.a.	5.38	10.43	n.a.	0.08	Moore (1984)[26]
MANOP S	K7905-54BC-1-M2	11.02°	140.09°	4905	bottom	0	1	n.a.	7.15	9.77	n.a.	0.09	
MANOP S	K7905-62BC-1-M2	11.03°	140.10°	4906	bottom	0	6.5	n.a.	11.88	29.38	n.a.	0.33	
MANOP S	K7905-66BC-1-M2	11.02°	140.10°	4906	bottom	0	1.7	n.a.	7.90	11.77	n.a.	0.10	
MANOP S	K7905-75BC-1-M2	11.02°	140.09°	4913	bottom	0	2.2	n.a.	2.28	9.27	n.a.	0.04	
MANOP S	K7905-91BC-1-M2	11.02°	140.10°	4908	bottom	0	6.5	n.a.	3.28	4.68	n.a.	0.05	
MANOP S	K7905-101BC-1-M2	11.02°	140.08°	4924	bottom	0	1.5	n.a.	5.00	10.82	n.a.	0.08	
MANOP S	K7905-104BC-1-M2	11.02°	140.09°	4914	bottom	0	6.1	n.a.	5.15	13.55	n.a.	0.27	
MANOP S	K7905-75BC	11.00°	140.00°	4900	top	0	0.093	0.10	13.88	5.68	0.80	0.15	Huh and Ku (1984)[16]
MANOP S	K7905-75BC	11.00°	140.00°	4900	top	0.093	1.64	0.10	7.37	4.62	0.15	0.17	

Site	Sample ID	Lat N	Long W	Water depth [m]	Nodule side	Depth min [mm]	Depth max [mm]	U-238 [Bq/g]	Th-230 [Bq/g]	Ra-226 [Bq/g]	Pa-231 [Bq/g]	Th-232 [Bq/g]	Data source
MANOP S	K7905-75BC	11.00°	140.00°	4900	top	1.64	2.35	0.13	1.50	1.77	0.05	0.22	
MANOP S	K7905-75BC	11.00°	140.00°	4900	top	2.35	2.85	0.08	0.00	0.96	0.01	0.00	
MANOP S	K7905-75BC	11.00°	140.00°	4900	top	2.85	5.85	0.10	0.11	0.23	0.02	0.13	
MANOP S	K7905-75BC	11.00°	140.00°	4900	top	5.85	8.85	0.06	0.07	0.13	0.01	0.06	
MANOP S	K7905-75BC	11.00°	140.00°	4900	top	8.85	12.8	0.05	0.14	0.13	0.02	0.06	
MANOP S	K7905-75BC	11.00°	140.00°	4900	bottom	0	0.056	n.a.	4.50	14.42	0.16	n.a.	
MANOP S	K7905-75BC	11.00°	140.00°	4900	bottom	0.056	0.122	n.a.	8.70	18.98	0.28	0.15	
MANOP S	K7905-75BC	11.00°	140.00°	4900	bottom	0.122	0.356	n.a.	5.78	20.85	0.12	0.32	
MANOP S	K7905-75BC	11.00°	140.00°	4900	bottom	0.356	1.23	n.a.	1.98	12.37	0.03	0.15	
MANOP S	K7905-75BC	11.00°	140.00°	4900	bottom	1.23	4.23	0.09	0.87	3.70	0.01	0.19	
MANOP S	K7905-75BC	11.00°	140.00°	4900	bottom	4.23	9.23	0.08	0.23	0.51	0.01	0.13	
MANOP S	K7905-75BC	11.00°	140.00°	4900	bottom	9.23	15.2	0.04	0.09	0.11	0.00	0.05	
MANOP S	K7905-47BC	11.00°	140.00°	4900	top	0	0.2	0.06	33.67	3.75	1.10	0.87	
MANOP S	K7905-47BC	11.00°	140.00°	4900	top	0.2	0.8	0.09	7.57	5.40	0.63	0.26	
MANOP S	K7905-47BC	11.00°	140.00°	4900	top	0.9	1.7	0.09	5.22	2.78	0.11	0.39	
MANOP S	K7905-47BC	11.00°	140.00°	4900	top	1.7	5.7	0.07	0.16	1.35	0.06	0.19	
MANOP S	K7905-47BC	11.00°	140.00°	4900	top	5.7	10.5	0.08	0.85	0.99	0.04	0.23	
MANOP S	K7905-47BC	11.00°	140.00°	4900	bottom	0	0.7	0.11	1.03	13.83	0.12	0.12	
MANOP S	K7905-47BC	11.00°	140.00°	4900	bottom	0.7	1.8	0.11	0.24	4.48	0.02	0.20	
MANOP S	K7905-47BC	11.00°	140.00°	4900	bottom	1.8	3.8	0.08	0.18	1.22	0.03	0.16	
MANOP S	K7905-47BC	11.00°	140.00°	4900	bottom	3.8	7.8	0.05	0.25	0.52	0.02	0.12	
MANOP S	K7905-47BC	11.00°	140.00°	4900	bottom	7.8	12.6	0.06	0.70	0.78	0.04	0.19	
MANOP R	RAMA 1-15BC	30.00°	158.00°	5800	top	0	0.19	0.19	63.62	4.57	0.83	1.12	
MANOP R	RAMA 1-15BC	30.00°	158.00°	5800	top	0.19	0.31	0.18	37.68	3.25	0.64	0.87	
MANOP R	RAMA 1-15BC	30.00°	158.00°	5800	top	0.31	0.49	0.14	18.62	3.12	0.14	1.04	
MANOP R	RAMA 1-15BC	30.00°	158.00°	5800	top	0.49	0.77	0.12	2.48	2.17	n.a.	0.86	
MANOP R	RAMA 1-15BC	30.00°	158.00°	5800	top	0.77	1.1	0.17	0.74	1.88	n.a.	0.94	
MANOP R	RAMA 1-15BC	30.00°	158.00°	5800	top	1.1	1.8	0.15	0.33	1.60	n.a.	0.72	

Site	Sample ID	Lat N	Long W	Water depth [m]	Nodule side	Depth min [mm]	Depth max [mm]	U-238 [Bq/g]	Th-230 [Bq/g]	Ra-226 [Bq/g]	Pa-231 [Bq/g]	Th-232 [Bq/g]	Data source
MANOP R	RAMA 1-15BC	30.00°	158.00°	5800	top	1.8	4.8	0.11	0.38	0.45	0.02	0.23	
MANOP R	RAMA 1-15BC	30.00°	158.00°	5800	top	4.8	9	0.12	0.00	0.21	n.a.		
MANOP R	RAMA 1-15BC	30.00°	158.00°	5800	top	9	13	0.12	0.28	0.05	n.a.	0.20	
MANOP R	RAMA 1-15BC	30.00°	158.00°	5800	bottom	0	0.05	n.a.	15.93	7.83	0.35	0.90	
MANOP R	RAMA 1-15BC	30.00°	158.00°	5800	bottom	0.05	0.1	n.a.	12.63	6.97	0.27	1.14	
MANOP R	RAMA 1-15BC	30.00°	158.00°	5800	bottom	0.1	0.18	0.17	7.98	6.53	0.05	1.38	
MANOP R	RAMA 1-15BC	30.00°	158.00°	5800	bottom	0.18	0.96	0.12	1.28	6.78	0.00	0.71	
MANOP R	RAMA 1-15BC	30.00°	158.00°	5800	bottom	0.96	3	0.08	0.14	0.67	n.a.	0.27	
MANOP R	RAMA 1-15BC	30.00°	158.00°	5800	bottom	3	7	0.00	0.18	0.16	n.a.	0.29	
MANOP R	RAMA 1-15BC	30.00°	158.00°	5800	bottom	7	11	0.18	0.16	0.26	n.a.	0.23	
MANOP R	RAMA 1-18BC	30.00°	158.00°	5800	top	0	0.04	0.18	45.68	2.92	2.73	1.29	
MANOP R	RAMA 1-18BC	30.00°	158.00°	5800	top	0.04	0.1	0.17	30.35	3.57	1.49	1.27	
MANOP R	RAMA 1-18BC	30.00°	158.00°	5800	top	0.1	0.3	0.15	13.58	2.33	0.32	1.16	
MANOP R	RAMA 1-18BC	30.00°	158.00°	5800	top	0.3	1.8	0.12	1.34	2.28	0.01	0.89	
MANOP R	RAMA 1-18BC	30.00°	158.00°	5800	top	1.8	5.3	0.12	0.12	0.54	0.01	0.21	
MANOP R	RAMA 1-18BC	30.00°	158.00°	5800	top	5.3	10	0.14	0.15	0.21	0.01	0.21	
MANOP R	RAMA 1-18BC	30.00°	158.00°	5800	bottom	0	0.18	0.17	41.63	8.83	2.25	0.88	
MANOP R	RAMA 1-18BC	30.00°	158.00°	5800	bottom	0.18	0.38	0.12	11.50	6.77	0.41	0.82	
MANOP R	RAMA 1-18BC	30.00°	158.00°	5800	bottom	0.38	2.9	0.08	0.12	1.26	0.01	0.21	
MANOP R	RAMA 1-18BC	30.00°	158.00°	5800	bottom	2.9	7	0.10	0.10	0.12	0.00	0.19	
MANOP R	RAMA 1-20BC	30.00°	158.00°	5800	top	0	0.04	0.15	43.45	3.05	1.78	1.20	
MANOP R	RAMA 1-20BC	30.00°	158.00°	5800	top	0.04	0.24	0.15	26.10	2.77	0.78	1.05	
MANOP R	RAMA 1-20BC	30.00°	158.00°	5800	top	0.24	1.2	0.11	4.03	2.12	0.09	0.64	
MANOP R	RAMA 1-20BC	30.00°	158.00°	5800	top	1.2	2.2	0.11	0.29	0.02	0.02	0.63	
MANOP R	RAMA 1-20BC	30.00°	158.00°	5800	top	2.2	6.2	0.07	0.09	0.22	n.a.	0.14	
MANOP R	RAMA 1-20BC	30.00°	158.00°	5800	top	6.2	10	0.09	0.11	0.11	0.01	0.26	
MANOP R	RAMA 1-20BC	30.00°	158.00°	5800	bottom	0	0.44	0.13	5.92	5.47	0.14	1.17	
MANOP R	RAMA 1-20BC	30.00°	158.00°	5800	bottom	0.44	0.9	0.12	0.57	5.32	0.03	0.97	

Site	Sample ID	Lat N	Long W	Water depth [m]	Nodule side	Depth min [mm]	Depth max [mm]	U-238 [Bq/g]	Th-230 [Bq/g]	Ra-226 [Bq/g]	Pa-231 [Bq/g]	Th-232 [Bq/g]	Data source
MANOP R	RAMA 1-20BC	30.00°	158.00°	5800	bottom	0.9	1.9	0.15	0.23	3.40	0.02	1.02	
MANOP R	RAMA 1-20BC	30.00°	158.00°	5800	bottom	1.9	4	0.09	0.27	1.02	0.00	0.32	
MANOP R	RAMA 1-20BC	30.00°	158.00°	5800	bottom	4	8	0.18	0.16	0.20	0.00	0.27	
MANOP R	RAMA 1-20BC	30.00°	158.00°	5800	bottom	8	12	0.16	0.26	0.18	n.a.	0.36	
MANOP H	PLUTO-3-6SBC	06.50°	093.00°	3570	top	0	3	n.a.	0.13	1.42	0.01	0.00	
MANOP H	PLUTO-3-6SBC	06.50°	093.00°	3570	top	3	6	0.07	0.34	2.00	0.01	0.01	
MANOP H	PLUTO-3-6SBC	06.50°	093.00°	3570	top	6	10	0.06	0.14	0.61	0.00	0.02	
MANOP H	PLUTO-3-6SBC	06.50°	093.00°	3570	bottom	0	1	0.06	0.29	2.05	0.02	0.01	
MANOP H	PLUTO-3-6SBC	06.50°	093.00°	3570	bottom	4	10	0.06	0.11	0.49	0.00	0.02	
MANOP H	VULCAN 1-378C	06.50°	093.00°	3570	top	0	5	0.05	0.58	2.00	0.01	0.01	
MANOP H	VULCAN 1-378C	06.50°	093.00°	3570	top	5	10	0.04	0.23	1.10	0.06	0.01	
MANOP H	VULCAN 1-378C	06.50°	093.00°	3570	top	10	14	0.05	0.18	0.44	0.01	0.01	
MANOP H	VULCAN 1-378C	06.50°	093.00°	3570	top	14	24	0.06	0.10	0.16	0.00	0.02	
MANOP H	VULCAN 1-378C	06.50°	093.00°	3570	bottom	0	3.5	0.08	0.17	0.54	0.01	0.00	
MANOP H	VULCAN 1-378C	06.50°	093.00°	3570	bottom	3.5	6	0.06	0.14	0.21	0.01	0.00	
MANOP H	VULCAN 1-378C	06.50°	093.00°	3570	bottom	6	8	0.03	0.13	0.18	0.01	0.00	
MANOP H	VULCAN 1-378C	06.50°	093.00°	3570	bottom	8	10	0.06	0.24	0.17	0.01	0.01	
MANOP H	VULCAN 1-378C	06.50°	093.00°	3570	bottom	10	14	0.07	0.13	0.14	0.01	0.00	
MANOP H	VULCAN 1-378C	06.50°	093.00°	3570	bottom	14	19	0.09	0.14	0.11	0.01	0.01	
MANOP H	VULCAN 1-378C	06.50°	093.00°	3570	bottom	0	3.5	0.08	0.17	0.54	0.01	0.00	
KODOS89-15	MN-89-15	09.33°	152.67°	5212	top	0	0.173	n.a.	10.50	n.a.	n.a.	n.a.	Moon et al. (1994)[55]
KODOS89-15	MN-89-15	09.33°	152.67°	5212	top	0.173	0.322	n.a.	3.80	n.a.	n.a.	n.a.	
KODOS89-15	MN-89-15	09.33°	152.67°	5212	top	0.322	0.425	n.a.	2.33	n.a.	n.a.	n.a.	
KODOS89-15	MN-89-15	09.33°	152.67°	5212	top	0.425	0.481	n.a.	0.65	n.a.	n.a.	n.a.	
KODOS89-15	MN-89-15	09.33°	152.67°	5212	bottom	0	0.075	n.a.	6.05	n.a.	n.a.	n.a.	
KODOS89-15	MN-89-15	09.33°	152.67°	5212	bottom	0.075	0.205	n.a.	6.35	n.a.	n.a.	n.a.	
KODOS89-15	MN-89-15	09.33°	152.67°	5212	bottom	0.205	0.285	n.a.	4.53	n.a.	n.a.	n.a.	
KODOS89-15	MN-89-15	09.33°	152.67°	5212	bottom	0.285	0.373	n.a.	2.15	n.a.	n.a.	n.a.	

Site	Sample ID	Lat	Long	Water depth	Nodule side	Depth min	Depth max	U-238	Th-230	Ra-226	Pa-231	Th-232	Data source
		N	W	[m]		[mm]	[mm]	[Bq/g]	[Bq/g]	[Bq/g]	[Bq/g]	[Bq/g]	
KODOS89-15	MN-89-15	09.33°	152.67°	5212	bottom	0.373	0.427	n.a.	1.98	n.a.	n.a.	n.a.	
KODOS89-15	MN-89-15	09.33°	152.67°	5212	bottom	0.427	0.546	n.a.	2.12	n.a.	n.a.	n.a.	

Table S3: Rn-222 emanation rates (E) determined for the different nodules (n=4) used for the experiment.

Measured nodule dimensions		Rn-222 emanation
d	h	E
[mm]	[mm]	[Bq cm^{-2} d^{-1}]
25	10	0.070
40	20	0.072
70	55	0.096
100	90	0.101

Table S4: Rn-222 concentrations [Bq L^{-1}] in the pore space after one half-life (3.8 days) storage, depending on the initial nodule size and the surface emanation rate (E). Here, we assume a range of nodule sizes with uniform eccentricity ($e = 0.6$) and min. and max. surface emanation rates of 0.07 and 0.101 Bq cm^{-2} d^{-1}, respectively, according to the results of the emanation experiment (cp. Table S3).

Nodule dimensions		Rn-222 concentration	
d	h	$E = 0.07$	$E = 0.101$
[mm]	[mm]	[Bq L^{-1}]	[Bq L^{-1}]
20	16	939	1354
30	24	626	903
40	32	469	677
50	40	375	542
60	48	313	451
70	56	268	387
80	64	235	339

Supplementary Figures

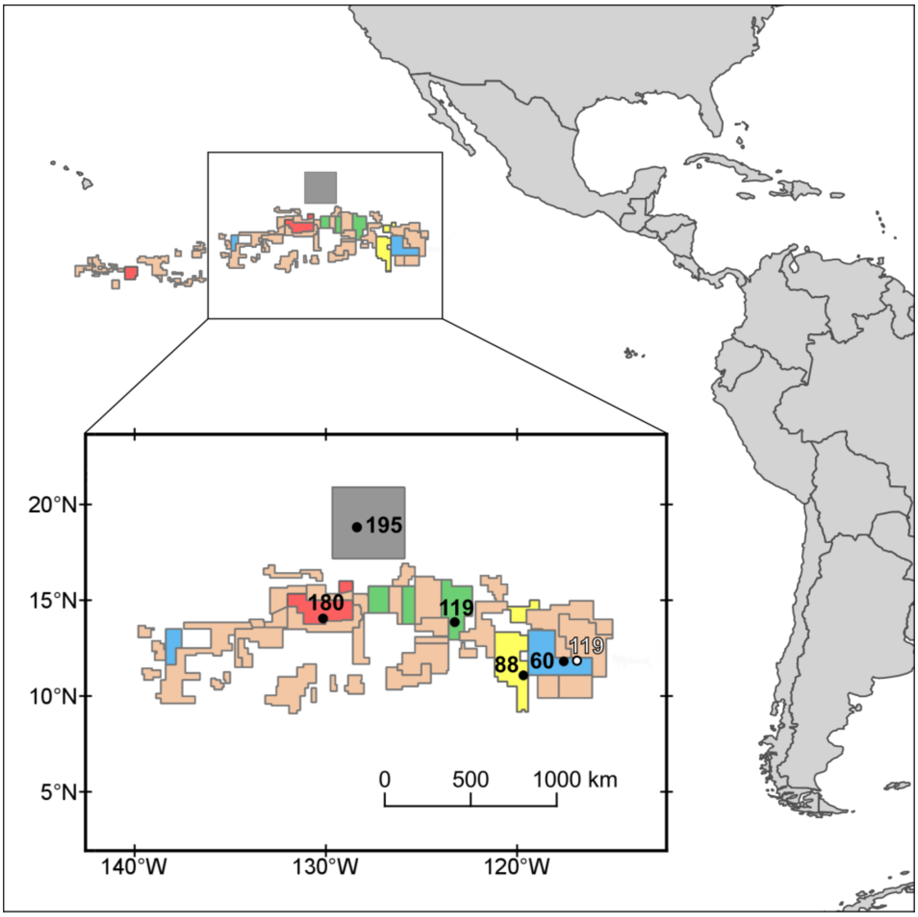

Fig. S1: Overview map of the Clarion-Clipperton Zone (CCZ) with different exploration areas for polymetallic nodules. Blue: German BGR (Bundesanstalt für Geowissenschaften und Rohstoffe/Federal Institute for Geosciences and Natural Resources) area; yellow: IOM (Interoceanmetal) Joint Organization area; green: Belgian GSR (Global Sea Mineral Resources NV) area; red: French IFREMER (Institut français de recherche pour l'exploitation de la mer) area; grey: Area of Particular Environmental Interest (APEI) No. 3, which is excluded of any mining activities. Black and white dots indicate the stations where the polymetallic nodules were retrieved during RV SONNE cruises SO239 and SO268, respectively. For detailed station information see Supplementary Table S1. Maps were created with QGIS3.4.4 'Madeira' using the map shapefile for the exploration areas provided by the ISA.

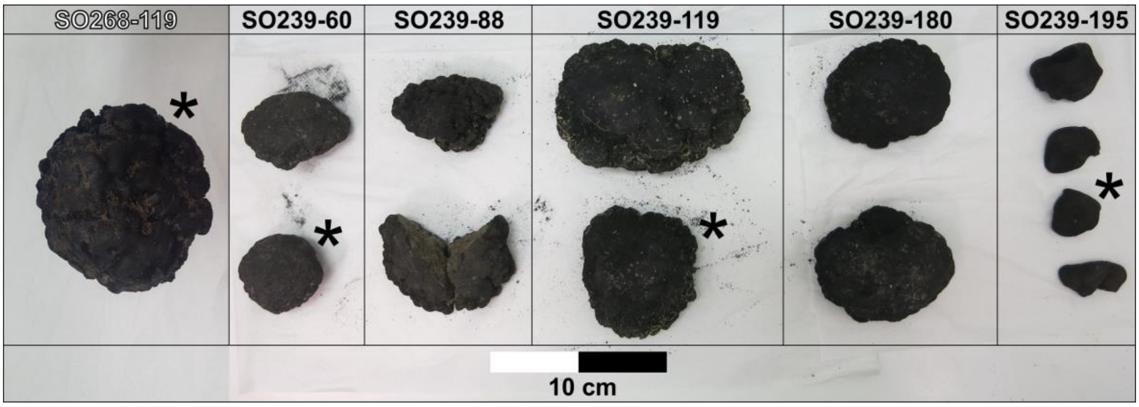

Fig. S2: Polymetallic nodules investigated in this study and retrieved during RV SONNE cruises SO239 and SO268 from different areas for the exploration of polymetallic nodules in the CCZ (see Supplementary Fig. S1). Nodules used for the measurement of Radon emanation are marked (*). For detailed station information see Supplementary Table S1.

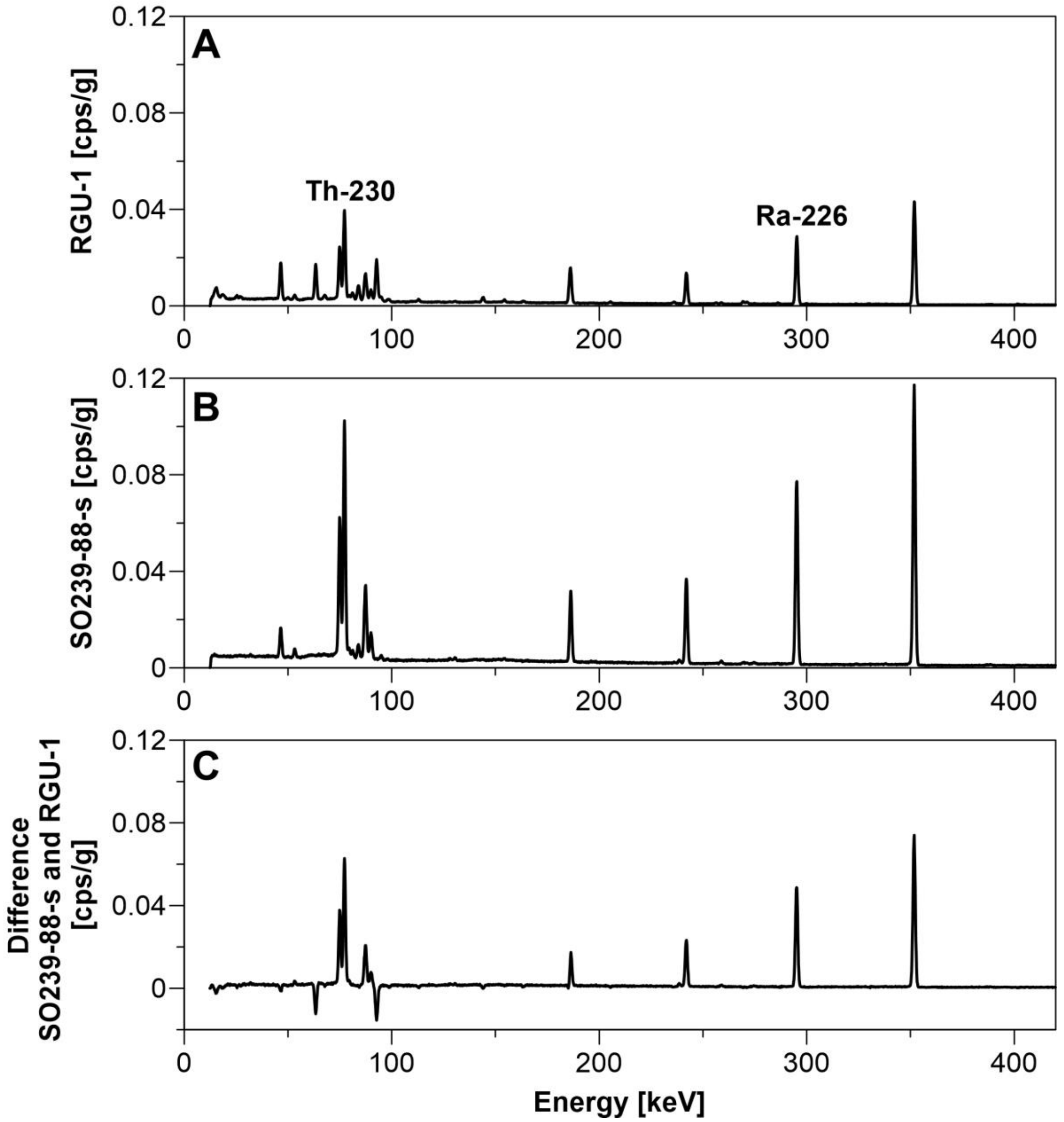

Fig. S3: Gamma spectra of (A) the uranium reference material RGU-1 (400 ppm uranium with daughters in full equilibrium) and (B) a sample from the surface of a polymetallic nodule from the IOM area (SO239-88-s, cp. Supplementary Table S1, Supplementary Fig. S2). (C) Direct comparison of the spectra of the surface nodule sample SO239-88-s and the RGU-1 reference material. Both gamma spectra were normalized to the same counting time and sample mass, and counted in the same detector. While some decay products indicating the presence of uranium in the sample are missing in the surface nodule sample SO239-88-s, uranium-series daughters in the nodule exceed the activity of the uranium ore sample. Activities up to five times higher have been reported previously for Th-230 on the surface of nodules[16], corresponding to the radiation emitted by uranium ores well in excess of 2000 ppm (0,2 % uranium by mass). For Pa-231, gamma line 402 keV was evaluated according to Geibert et al. (2001)[56] but the peak is barely visible in the spectrum.

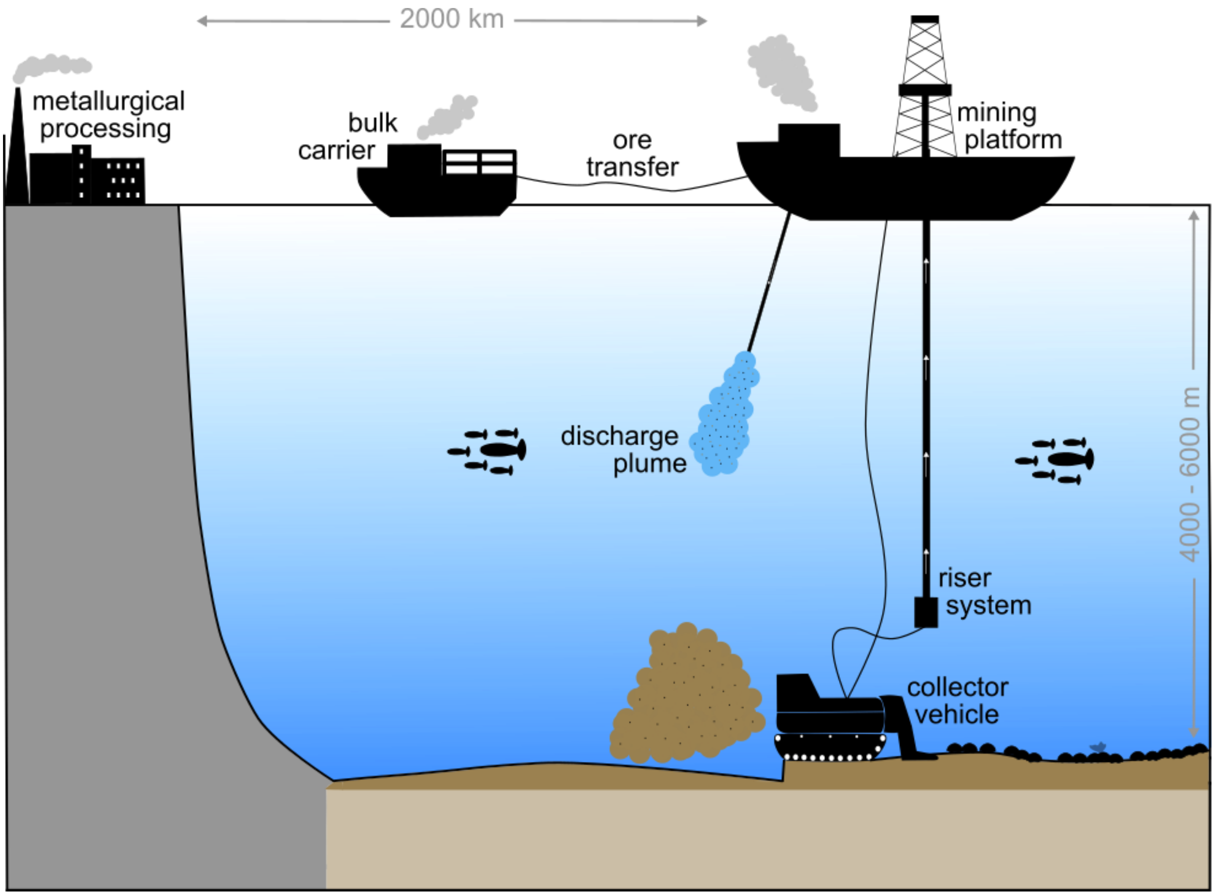

Fig. S4: Anticipated components of an industrial deep-sea polymetallic mining setup. A commercial mining system for polymetallic nodules is expected to consist of a nodule collector vehicle at the seafloor, which is connected to a mining platform at the sea surface via a vertical riser system, a discharge system at some depth in the water column and an ore transfer from the mining platform to a bulk carrier for the transport of ore to the shore-based metallurgical processing plant[2,39]. Residuals from the mining slurry are expected to include bottom seawater, sediments and nodule fines, which are released back into the ocean as a discharge plume[2,39]. Modified after Hein et al. (2020)[2].

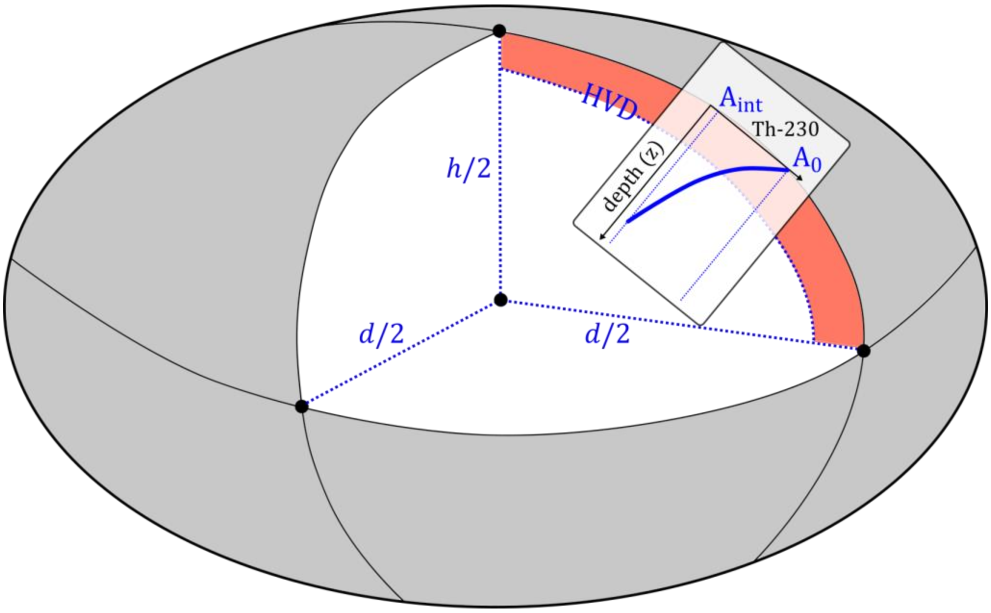

Fig. S5: Illustration of a spheroid-shaped nodule as basic assumption for the calculation of the bulk nodule Th-230 activity. The calculation includes the nodule height (h), equatorial diameter (d), Th-230 activity at the nodule surface (A_0), Th-230 activity in the interior of the nodule (A_{int}), half-value depth (HVD) and the dry bulk density (DBD). The Th-230 activity decreases from high values in the outer nodule layer (red, thickness exaggerated for clarity) exponentially with depth z into the interior of the nodule. The Th-230 activities chosen for A_0 and A_{int} are extracted from new nodule radioisotope data presented as part of this study and from published datasets (Fig. 2; Supplementary Table S2). The calculation is publicly accessible with adjustable input parameters via the web application: https://jevolz.shinyapps.io/Nodule_alpha_radiation/.

Supplementary References

54. Kadko, D. & Burckle, L. H. Manganese nodule growth rates determined by fossil diatom dating. *Nature* **287(5784)**, 725–726 (1980). https://doi.org/10.1038/287725a0
55. Moon, D. S., Kim, K. H. & Kang, J. K. Uranium-Series Growth Rates of Two Manganese Nodules from the KODOS-89 Site, Clarion-Clipperton Fracture Zone of the Central Equatorial Pacific. *J. Korean. Soc. Oceanogr.* **29(3)**, 248–257 (1994).
56. Geibert, W. Actinium-227 als Tracer für Advektion und Mischung in der Tiefsee. *Ber. Polarforsch. Meeresforsch.* **385** (2001).
